# Rapid and Significant Angioarchitectural Changes in the Carotid Artery and Its Branches After Endovascular Treatment of Acute Hemorrhage Due to a Cerebral Arteriovenous Malformation

**DOI:** 10.3390/biomedicines12122704

**Published:** 2024-11-26

**Authors:** Adam Dobek, Wojciech Szubert, Katarzyna Kurzyk, Karol Zaczkowski, Karol Wiśniewski, Ludomir Stefańczyk

**Affiliations:** 1 Department of Radiology and Diagnostic Imaging, Norbert Barlicki Memorial Teaching Hospital No. 1, Medical University of Lodz, 90-153 Lodz, Poland; 2 Department of Neurosurgery and Neurooncology, Norbert Barlicki Memorial Teaching Hospital No. 1, Medical University of Lodz, 90-153 Lodz, Poland

**Keywords:** angioarchitecture, cerebral arteriovenous malformation, carotid artery, embolization, hemorrhage

## Abstract

Introduction: Cerebral arteriovenous malformations (CAVMs) are rare, with an estimated prevalence of 0.01%. Symptoms typically present in adults under 40, often beginning with hemorrhage in 61% of cases. The annual risk of hemorrhage is between 2–4%, with a mortality rate of 10%, and 50% of survivors may experience permanent neurological deficits. Embolization can induce changes in the angioarchitecture of the affected vessels. Our case uniquely demonstrates a morphological alteration in the carotid artery (CA) and its primary branches, associated with the presence of a CAVM. Detailed Case Presentation: A 52-year-old patient presented to the Emergency Department with weakness, shortness of breath, dizziness, nausea, and vomiting that began earlier that morning. Clinical and radiological evaluations suggested acute bleeding from a ruptured CAVM. The patient was admitted for digital subtraction angiography (DSA) and potential embolization. DSA confirmed the presence of a CAVM, supplied by the middle meningeal and occipital arteries, with distortion of the left CA angioarchitecture. Selective catheterization and embolization using Onyx 18 were successfully performed. After receiving supportive treatment, the patient was discharged in good condition. A follow-up CT scan three months later showed complete resolution of the brain pathologies. A repeat angiogram revealed no recanalization of the CAVM, with normalization of the CA and its branches. The patient is currently asymptomatic. Conclusions: Detection of pathological changes in the head and neck arteries linked to CAVM can predict rupture risk and complicate endovascular access. Identifying these changes early warrants consideration of interventions to prevent hemorrhage, though alternative access routes or strategies may be needed for safe, effective treatment.

## 1. Introduction

### 1.1. Characteristics of CAVM and Its Clinical Impact

Cerebral arteriovenous malformations (CAVMs) are rare developmental abnormalities characterized by clusters of pathological vessels, where arterial supply vessels are directly connected to drainage vessels within the venous system [1,2]. The estimated prevalence of CAVMs in the general population is extremely low, approximately 0.01% [3]. Many individuals with CAVMs are asymptomatic, which may lead to an underestimation of this figure. However, when CAVMs become symptomatic, this typically occurs in young adults, often before the age of 40. Hemorrhage is the most common initial symptom of a ruptured fistula, occurring in approximately 61% of cases. Other symptoms may include non-specific headaches, altered consciousness, nausea, vomiting, dizziness, seizures, balance disturbances, as well as speech and vision impairments [4,5,6]. The annual risk of hemorrhage from an unruptured CAVM ranges between 2% and 4%, with associated morbidity rates of 20–50% and a mortality rate of approximately 10% [7]. Additionally, the risk of rebleeding within one year after an initial hemorrhage can be as high as 33%, with up to 50% of cases resulting in permanent neurological deficits [2,6]. These statistics underscore the importance of early identification of CAVMs and prompt planning of therapeutic management to mitigate these risks.

### 1.2. Therapeutic Options and Implications of Endovascular Treatment for CAVM

Current treatment options for CAVMs include microsurgical resection, stereotactic radiosurgery, and endovascular embolization, either individually or in combination. The choice of treatment depends on several factors, including the lesion’s angioarchitecture (e.g., size of the nidus, presence of feeding arteries or nidal aneurysms, and large arteriovenous fistulas), lesion location, and the expertise of the treating center. Microsurgical resection is considered the gold standard for accessible lesions, particularly in cases where bleeding has occurred and hematoma evacuation is feasible. Stereotactic radiosurgery is typically reserved for lesions smaller than 3 cm that are surgically inaccessible, although its efficacy diminishes for larger lesions. Endovascular embolization is often used as an adjunct to surgery or radiosurgery, with the aim of reducing hemorrhage risk or decreasing the lesion size prior to radiation. In select cases, embolization alone can achieve success rates exceeding 90%, particularly for small to medium-sized, superficial lesions with a compact nidal architecture, supplied by one or two arterial feeders, and drained by a well-delineated venous network [3,8,9]. The management of CAVMs, however, remains a topic of ongoing debate. In cases of ruptured CAVMs, intervention is generally recommended to prevent re-hemorrhage. Conversely, the ARUBA trial found that patients undergoing interventional treatment had a higher risk of bleeding or death compared to those managed conservatively. These findings have sparked significant discussion and remain a point of contention within the field [6,8,9,10].

### 1.3. Clinical Case Presentation: A Novel Finding in CAVM Diagnosis and Management

A notable phenomenon observed following embolization is the alteration in the angioarchitecture of vessels involved in CAVMs. Buell et al., in their review, highlighted that embolization of CAVMs can induce angiogenesis, identifying three primary mechanisms underlying this process: (1) hypoxia-mediated angiogenesis, (2) inflammation-mediated angiogenesis, and (3) hemodynamic-mediated angiogenesis [11]. Similarly, other researchers report that post-treatment changes in the angioarchitecture of CAVMs occur in approximately 40% of cases [5,12]. In contrast, morphological changes in the large vessels of the head and neck associated with arteriovenous malformations are exceedingly rare. Notably, such changes have only been reported in cases where the malformations do not involve cerebral tissue [13]. Our case uniquely demonstrates a morphological alteration in the carotid artery (CA) and its primary branches associated with the presence of a CAVM, with normalization of vessel morphology following successful closure of the CAVM. To our knowledge, this is the only documented case in the world literature illustrating such a change, wherein the presence of a CAVM influences the morphology of the CA.

## 2. Detailed Case Description

### 2.1. Clinical Presentation and Imaging Findings

**Figure 1:**
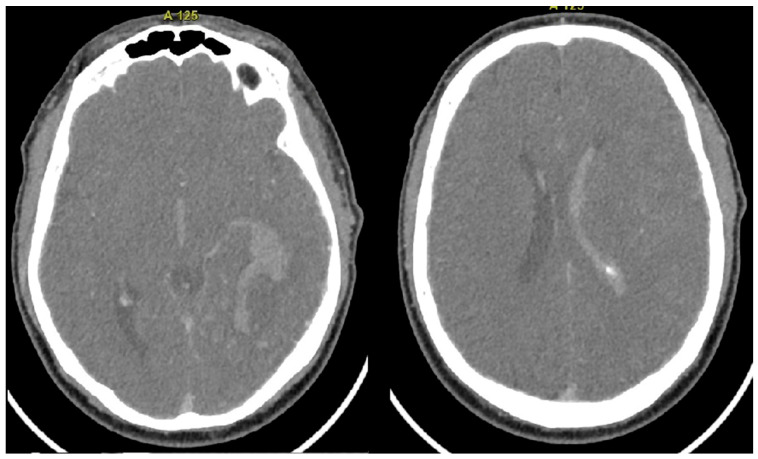
Computed Tomography (CT) Images, Axial View: The images reveal acute intracerebral and intraventricular hemorrhage.

**Figure 2:**
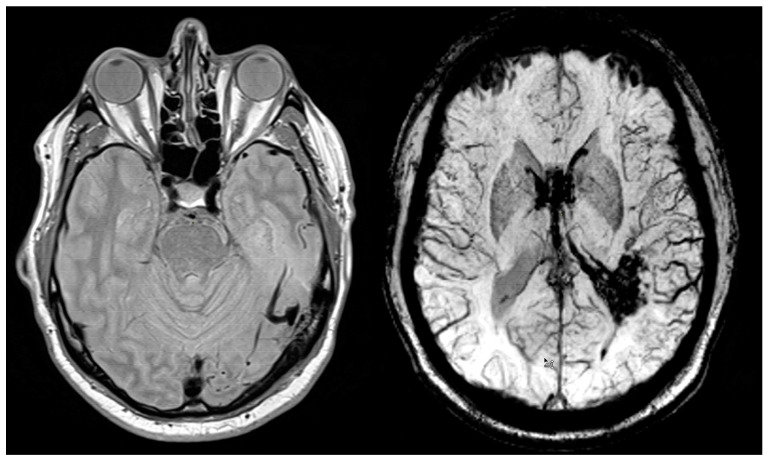
Magnetic Resonance Images, Axial View: The images reveal a cerebral arteriovenous malformation with tortuous vessels. Proton density sequence T2-weighted susceptibility-weighted imaging sequence.

A 52-year-old patient presented to the Emergency Department with symptoms of generalized weakness, shortness of breath, dizziness, nausea, and vomiting, all of which began earlier in the day. The patient had no significant medical history. Neurological and ophthalmological consultations were requested, alongside imaging and laboratory tests. On neurological examination, the patient was alert but exhibited psychomotor slowing. A positive left-sided Kernig’s sign was noted, as well as asymmetry in pupil size (right > left), although both pupils remained reactive to light. No pathological reflexes or motor paresis were observed. Ophthalmological evaluation revealed right-sided homonymous visual field defects. Laboratory tests showed an elevated D-dimer level of 1000 ng/mL. Head CT imaging demonstrated a hematoma in the left occipital lobe, surrounded by hypodense edema extending into the ventricular system. Additionally, a hyperdense area was identified in the transverse sinus, along with findings of reduced pericerebral fluid reserve and a slight midline shift (Figure 1). MRI further revealed tortuous vessels with dilated draining veins near the left jugular bulb and left temporal lobe, consistent with findings suggestive of a CAVM (Figure 2).

### 2.2. Confirmation of CAVM Diagnosis Through DSA with Interventional Treatment, and Concurrent Left Carotid Artery and Its Branches Pathologies

**Figure 3:**
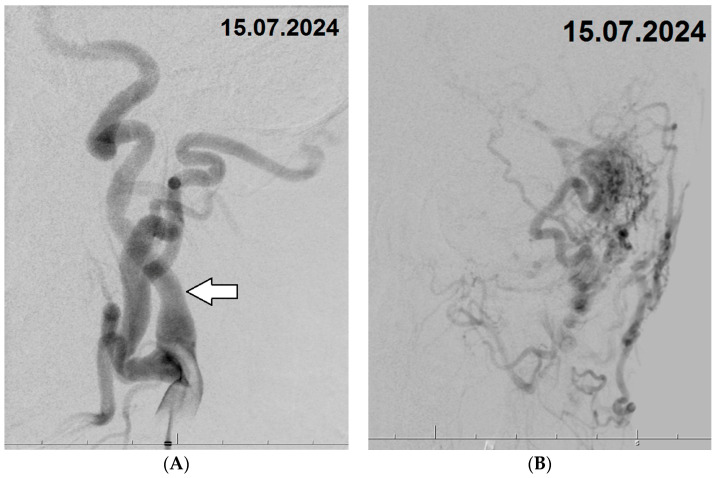
(**A**,**B**) Digital Subtraction Angiography (DSA) Images Pre-Embolization: (**A**)—Image of the carotid artery (CA) along with its thickened and tortuous branches (white arrow indicating the internal CA). (**B**)—Image of the cerebral arteriovenous malformation (CAVM), displaying its feeding and draining vessels.

The patient was subsequently admitted to the neurosurgery department and scheduled for digital subtraction angiography (DSA) with a potential embolization procedure. Under general anesthesia, vascular access was obtained through the right femoral artery using a 6F vascular sheath. Selective catheterization was performed on both internal carotid arteries (ICAs), both external carotid arteries (ECAs), and the left vertebral artery. The branches of the left CA appeared pathologically tortuous and significantly thickened, with diameters of 5.3 mm for the ICA and 6.2 mm for the ECA. Angiograms confirmed the presence of a cerebral arteriovenous malformation (CAVM) in the left temporal region. The CAVM was primarily supplied by branches of the middle meningeal and occipital arteries originating from the left ECA, with venous drainage into cortical veins, the superior petrosal sinus, and the sagittal sinuses (Figure 3A,B). Following a detailed evaluation of the vascular anatomy, selective catheterization of the distal middle meningeal artery was performed using an Apollo 1.5 microcatheter. Embolization of the CAVM was successfully completed with the use of two ampoules of Onyx 18. A follow-up angiogram demonstrated significant closure of the CAVM, with only minimal residual contrast filling from branches of the occipital artery. The vascular access site was then closed using an Angio-Seal device.

### 2.3. Hospitalization Summary and Immediate Post-Treatment Outcome

After mobilization, the patient was discharged in good general and neurological condition. The patient was fully alert, oriented, and exhibited no signs of paresis. During the hospitalization, the patient received comprehensive care, including fluid therapy, pain management, antiepileptic treatment, and therapy to address cerebral edema. A follow-up angiography was scheduled for three months post-discharge.

### 2.4. Follow-Up Evaluation at Three Months Post-Intervention

**Figure 4:**
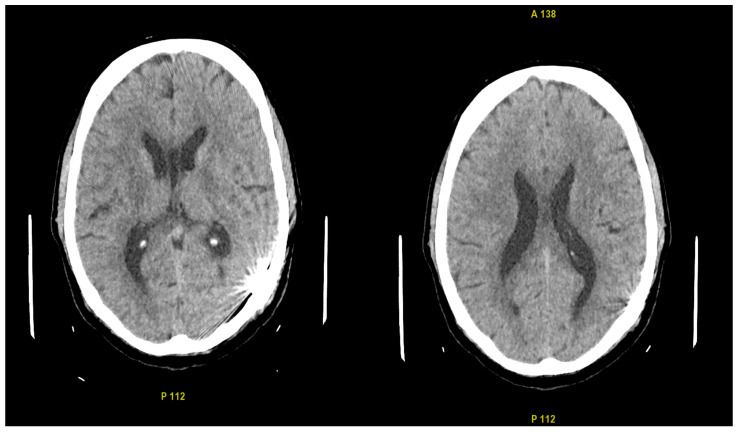
CT Images, Axial View (3 Months Post-Embolization): The images demonstrate normalization of brain morphology, with embolization artifacts visible in the region of the left occipital lobe.

**Figure 5:**
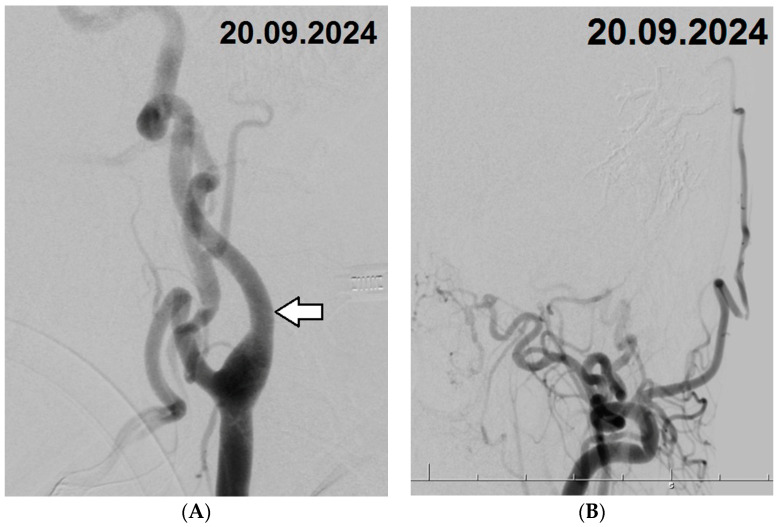
(**A**,**B**) DSA Images Approximately 3 Months Post-Embolization (**A**)—Image shows the CA and its branches, with normalized angioarchitecture (white arrow indicating the internal CA). (**B**)—Image confirms the complete embolization of the CAVM.

Approximately three months later, the patient was readmitted, and a CT scan revealed complete resolution of the previously identified pathologies (Figure 4). A follow-up digital subtraction angiography (DSA) was performed under general anesthesia via the left femoral artery. Using a 6F sheath, selective catheterization of both ICAs, both ECAs, and the left vertebral artery was conducted, with no evidence of fistula recanalization. The access site was again closed using an Angio-Seal device, and the procedure proceeded without complications. Notably, comparison with the initial DSA demonstrated significant changes in the angioarchitecture of the CA branches. These changes included marked straightening and a reduction in vessel diameters, which now measured 4.9 mm for the ICA and 4.2 mm for the ECA (Figure 5A,B). After an uneventful postoperative course, the patient was mobilized and discharged home in good general condition. During hospitalization, the patient received fluid therapy, pain management, and antiepileptic treatment. The patient is currently asymptomatic and has returned to baseline functioning, as before the hemorrhagic incident.

## 3. Discussion

This case study presents a unique situation where, in addition to the CAVM, pathological changes in the morphology of the CA were observed on the same side as the malformation. The CA demonstrated marked tortuosity and significant thickening, along with its main branches, including the ECA and ICA. Following successful embolization and closure of the CAVM, normalization of the CA morphology and its branches was observed during the 3-month follow-up. This finding suggests a direct relationship between the presence of the CAVM and the morphological changes in the CA and its branches. To date, such vascular changes associated with CAVMs have not been reported in the literature. For instance, Hou et al. described a case with a CAVM morphology similar to ours; however, no morphological changes in the large arteries were observed. Furthermore, they noted that the main blood supply to CAVMs typically arises from the ICA and vertebral arteries, emphasizing the atypical nature of the lesions we describe [14]. In the literature, isolated cases have been reported where head and neck malformations caused changes in the morphology of large arteries in this region, but these changes were not explored in detail. In a case series presented by Dawkins et al., two cases demonstrated such changes. In the first, massive dilation of the facial artery supplying an arteriovenous malformation was noted. Following successful embolization, DSA performed three months later revealed normalization of the vessel. In the second case, significant dilation of the ophthalmic artery associated with a palpebral arteriovenous malformation was described. However, the impact of embolization on this vascular structure was not discussed [13]. Conversely, embolization-induced angiogenesis and associated angioarchitectural changes in the periphery of CAVMs are well-documented. These changes occur in up to 40% of cases. Quarta Colosso et al. defined angioarchitectural modifications of CAVMs as any changes in the supply arteries, nidus, or draining veins observed between follow-up DSA studies, classifying them as either progression or regression. Progression events included the emergence of feeder arteries, recanalization of occluded feeders, recruitment of meningeal or dural arteries, the appearance of proximal or pedunculated aneurysms, increased nidus volume, enlargement of draining veins, or the development of focal venous ectasias [5]. Many researchers are investigating the impact of such changes on treatment outcomes and brain tissue perfusion. For example, Nocuń et al. analyzed brain tissue perfusion using SPECT imaging before and after CAVM embolization. Hypoperfusion was identified in the CAVM area in 17 cases and in distal brain regions in 12 cases before embolization. Post-embolization, perfusion around the CAVM worsened in 11 cases and improved in 3 (mainly in large CAVMs), while distal regions showed worsened perfusion in 9 cases and improvement in 3 [15]. Similarly, Shellikeri et al. used color-coded quantitative DSA to analyze peri-nidal peak velocity rates, referencing the ICA and vertebral artery. Post-embolization, 14 out of 19 cases demonstrated significant increases in perfusion, with no correlation between lesion size and peri-nidal perfusion. Notably, no significant changes were observed in tissue distant from the CAVM, although flow in draining veins slowed significantly post-embolization [16]. Markl et al. employed 4D flow MRI to analyze blood flow changes after embolization, observing reduced peak velocities and overall flow in arterial feeders, indicating a decreased blood supply to the CAVM nidus post-treatment [17]. These results, however, remain contradictory, likely due to methodological differences. Cerebral hemorrhage is a frequently reported complication following CAVM embolization. Spetzler et al. proposed that high-flow, low-resistance CAVMs cause chronic vasodilation and loss of vasomotor tone. Following embolization, normalization of perfusion pressure occurs, but impaired autoregulatory capacity may fail to manage the sudden pressure increase, leading to hemorrhagic events [18]. According to the latest report by the Society of NeuroInterventional Surgery Standards and Guidelines Committee, bleeding complications occur in approximately 6.5% of patients undergoing embolization [8]. Furthermore, literature reports suggest that pressure changes associated with dural CAVMs can induce new vascular anomalies or render CAVMs clinically dormant due to decreased local arterial pressure. Sudden pressure increases, however, can trigger hemorrhagic incidents [19,20]. In our case, during the acute hemorrhagic phase, the CA and its branches were dilated and tortuous, but they returned to normal after treatment. This observation indicates a direct link between the presence of the CAVM and the vascular alterations. Based on the experiences reported by other authors, these changes are likely related to hemodynamic alterations induced by the CAVM. It is worth considering whether such CA artery and its branches changes could serve as potential predictors of hemorrhagic risk in CAVM patients. The observed dilation and tortuosity during the acute phase may reflect an increased risk of rupture. Additionally, the interventional radiologist performing the procedure reported significant difficulties navigating the abnormally tortuous vessels, complicating catheter advancement to the lesion. The primary limitation of this manuscript is that the phenomenon described is based on a single case. To derive clinically significant conclusions, a larger study cohort is required. Given the exceptional rarity of this phenomenon, a multicenter study would likely be necessary to assemble a sufficiently robust dataset. Such an approach could enable better validation of the observed vascular morphological changes and their potential clinical implications.

## 4. Conclusions

The detection of pathological changes in the angioarchitecture of the large arteries in the head and neck associated with CAVM presence may serve as a positive predictor for the risk of CAVM rupture. Furthermore, these changes can significantly complicate endovascular access to the vessels supplying the CAVM. Therefore, when such alterations are identified, early consideration of potential interventional treatment to prevent hemorrhage is warranted. However, due to the potential challenges in accessing the malformation, alternative vascular access routes or other therapeutic strategies should be explored to ensure both the safety and efficacy of the treatment.

## Data Availability

The data presented in this study are available on request from the corresponding author.
